# Do Morphological Changes in the Anterior Mandibular Region Interfere with Standard Implant Placement? A Cone Beam Computed Tomographic Cross-Sectional Study

**DOI:** 10.1155/2020/8861301

**Published:** 2020-12-18

**Authors:** Shaimaa M. Fouda, Passant Ellakany, Marwa Madi, Osama Zakaria, Fahad A. Al-Harbi, Maha El Tantawi

**Affiliations:** ^1^Department of Substitutive Dental Sciences, College of Dentistry, Imam Abdulrahman Bin Faisal University, P.O. Box 1982, Dammam 31411, Saudi Arabia; ^2^Department of Preventive Dental Sciences, College of Dentistry, Imam Abdulrahman Bin Faisal University, P.O. Box 1982, Dammam 31411, Saudi Arabia; ^3^Department of Biomedical Dental Sciences, College of Dentistry, Imam Abdulrahman Bin Faisal University, P.O. Box 1982, Dammam 31411, Saudi Arabia; ^4^Department of Pediatric Dentistry and Dental Public Health, Faculty of Dentistry, Alexandria University, P.O. Box 21527, Alexandria, Egypt

## Abstract

**Objective:**

To determine the morphological features in the anterior mandibular region, the presence of lingual foramen and canal dimensions in Saudi subjects that would interfere with standard implant placement.

**Methods:**

CBCT scans of patients seeking implant treatment were examined. Based on the dentition status, patients were categorized into edentulous (group I) and dentulous (group I). On the panoramic view, the distance between the two mental foramina was divided into vertical segments of 10 mm width. In each segment, vertical bone height and buccolingual thickness at three levels (alveolar crest, 5 mm, and 10 mm apical to the crest) were assessed. The lingual foramen prevalence and canal features were assessed as well. Comparisons between the two groups regarding the assessed parameters were performed using the *t*-test. The percentage of edentulous mandibles with thickness <6 mm corresponding to the standard implant diameter was also calculated.

**Results:**

Following the inclusion and exclusion criteria, group I consisted of 45 subjects and group II comprised 26 subjects. Bone height and thickness at the crestal level were significantly less in edentulous (I) than dentate mandibles (II) (*P* < 0.0001). The lingual foramen was detected in 90% of patients. In both groups, males had significantly greater mandibular height than females (*P*=0.02 and 0.005). At the crestal level, the thickness was <6 mm in 50% of the anterior mandibular segments.

**Conclusion:**

Half of the edentulous patients may receive normal size implants in the anterior interforaminal segments, while the other half will be limited to narrow implants (3.5 mm and less). The lingual foramen location, canal size, and position may represent another limitation for implant placement in that segment.

## 1. Introduction

Tooth loss results in partial or complete edentulousness. The percentage of edentulous patients is still high in many countries even with all the attempts to reduce tooth loss [1]. Conventional removable complete or partial dentures are commonly used to restore missing teeth. Reduced retention and stability of conventional mandibular denture are frequently seen in patients with significant ridge resorption [2].

During the last few years, the use of implants as a treatment option in oral rehabilitation has increased dramatically [3]. Therefore, hybrid solutions and implant-supported overdenture may be a suitable treatment choice for patients having edentulous mandibles [2, 4].

However, treatment planning, placement, and restoration of dental implants for dentate and edentulous patients can be challenging. Anatomical limitations can make the identification of the correct location to place implants difficult. Insufficient bone height or width and the approximation to mental or lingual foramina are obstacles that face clinicians when placing an implant in the anterior mandibular region [5, 6].

Cone beam computed tomography (CBCT) is widely used nowadays in dental treatment particularly in planning for dental implants [5]. Also, guided implant surgery utilizes the CBCT images to plan and place implants in areas with a close approximation to anatomical structures [7]. CBCT overcomes the superimposition, distortion, and magnification errors of two-dimensional panoramic and intraoral radiographs [8]. CBCT provides three-dimensional images with high resolution and accuracy that can be seen from various angles with less radiation dose and cost than computed tomography [9]. Previous investigations assessed the safe zone in relation to the mental foramen, incisive canal, anterior loop of mental nerve, genial tubercle, and bone height and width [9–11].

The lingual foramen is located lingually in the midline of the mandibular symphysis at the level of the genial tubercle or superior to it [12]. Variations in the existence, number, and dimensions of the lingual canal have been reported [13, 14]. Special care must be taken during implant placement in the mandibular symphysis area to avoid injury to the lingual canal that would result in severe bleeding due to the presence of the neurovascular bundle [15] and to avoid perforation of the cortical plate. Injury to the inferior alveolar nerve and the lingual nerve is the most common complication in oral surgery procedures [16, 17]. This could lead to permanent neurosensory disturbance to the lower lip. Complications such as loss of lip and chin sensation may result in lip biting, impaired speech, and diminished salivary retention [15, 18, 19].

To the best of the authors' knowledge, the present study was the first to investigate the effect of morphological changes and features including lingual foramen and canal's dimensions in the Saudi population using CBCT.

The aim of this cross-sectional study was to assess morphological features in the mandibular interforaminal region, the presence of lingual foramen and canal dimensions in dentate and edentulous Saudi patients, and its influence on future implant planning.

## 2. Methods and Materials

This cross-sectional study involved CBCT images taken for the patients who visited the periodontology and prosthodontics dental clinic of IAU from January 2018 to January 2019 for dental implant treatment. Ethical approval was obtained by the Research Ethics committee (#2017019). The CBCT scans were taken in accordance with the standard protocol for the purpose of diagnosis and treatment planning of dental implant rehabilitation. Based on the dentition status of patients, groups identified in the study were edentulous (I) and dentulous (II). Written informed consent for participation was taken for this study in accordance with the national legislation and the institutional requirements. Initially, a total of 150 scans were collected. The current study was conducted in accordance with the Strengthening the Reporting of Observational Studies in Epidemiology (STROBE) guidelines [20].

Inclusion criteria were patients who visited the dental clinics seeking implant treatment and subjected to CBCT scan with complete patients' information. Acceptable CBCT images show both arches. For group I, patients with complete edentulous mandible. For group II, patients with complete dentulous mandible.

Exclusion criteria were patients <18 years old, patients with systemic disease or taking medications that affect bone turnover, incomplete patients' record, partial CBCT scans, and presence of artifacts or bone pathology. Following the inclusion and exclusion criteria, group I comprised of 26 subjects and group II comprised of 45 subjects.

All scans were taken using the KODAK 9500 Cone Beam 3D System (Carestream, Rochester, NY, USA) with a flat panel detector. The imaging area was a cylinder of 15–20.6 cm height and 9–18 cm diameter. Standard resolution mode (voxel size of 0.2 mm) was selected.

The standard exposure parameters were set to 90 kV tube voltage, 10 mA tube current, and 10.8 s exposure time. Examination was performed by 360° rotation in the occlusal position with the patient standing and closing their jaws. CS 3D Imaging Software (3.4.3. Carestream Health Inc. Atlanta, USA) was used for the evaluation of the CBCT obtained DICOM, and the full volumes were assessed.

**Figure 1:**
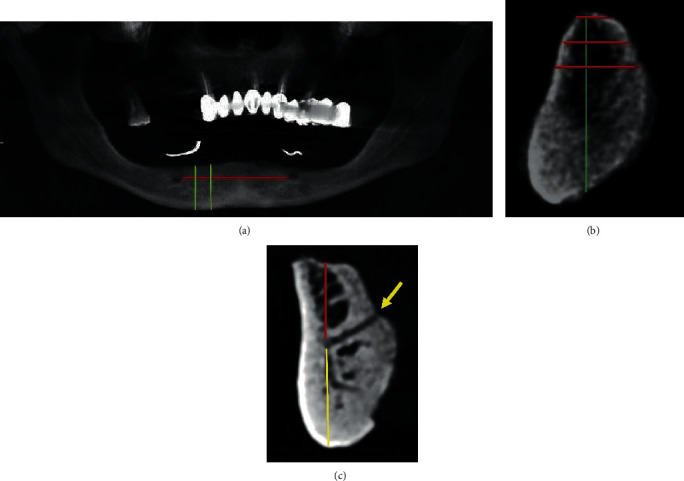
(a) Panoramic view of intermental foramina distance. (b) Vertical bone height in a cross-sectional view and buccolingual thickness at three levels in a cross-sectional view. (c) Red line, distance from the alveolar crest to the base of the lingual canal. Yellow line, distance from the base of the lingual canal to the base of mandible. Arrow shows the orifice of lingual foramen and length of the lingual canal from the orifice to the base of the lingual canal.

The software provides panoramic, axial, and cross-sectional images in the same screen. On the panoramic image, the distance between the two mental foramina was measured and divided horizontally into vertical segments of 10 mm width parallel to the midsagittal plane starting from the mesial side of the right mental foramen to the left side (Figure 1(a)) [21]. The distance of 10 mm was selected to accommodate the average implant diameter used in the mandibular area (4–7 mm).

### 2.1. The Radiographic Assessment

In each segment, vertical bone height was measured as the distance from the alveolar crest to the base of the mandible on the cross-sectional view of the axial plane using the ruler measuring tool of the software (Figure 1(b)). On the same view, mandibular buccolingual thickness was measured at three different levels: level A: at the alveolar crest, level B: 5 mm apical to the crest, and level C: 10 mm apical to the crest (Figure 1(b)). The presence of the lingual foramen was assessed in the middle segment (between the two mental foramina) in between the mandibular central incisors. The distances from the base of the lingual canal to the crest of alveolar bone and to the base of the mandible were measured as shown in Figure 1(c). The length of the canal was measured from the orifice of lingual foramen to the base of the lingual canal in the same segment horizontally.

Image analysis was carried out by two independent examiners. The first examiner performed vertical and horizontal measurements and the second performed the lingual foramen measurements. All measurements were reassessed after one month by the same precalibrated examiner with over 6 years of experience with CBCT on high-quality monitors in a room with dimmed light. The two sets of measurements compared using intraclass correlation coefficient values of at least 0.9 were obtained. The average of the two measurements was used for statistical analysis.

### 2.2. Statistical Analysis

Comparisons between dentulous and edentate patients and between males and females in mandibular thickness, height, and lingual features were performed using the *t*-test. Comparing the number of segments between the two groups was performed using chi square. Percentiles of mandibular thickness at levels *A* and *B* were calculated to assess the percentages of edentulous mandibles with thickness <6 mm corresponding to the standard thickness of implants in this region. Statistical analysis was performed using SPSS version 22.0. Significance level was set at 5%.

## 3. Results

The study included 71 CBCT scans of 26 edentulous (group I) and 45 dentate (group II) mandibles of patients of mean (SD) age in years = 59.0 (13.0) and 47.7 (12.7) with a statistically significant difference in age (*P*=0.001). Females were 13 (50%) in the edentulous group and 29 (64.4%) in the dentate group with no significant difference between groups in sex distribution (*P*=0.23).

**Figure 2:**
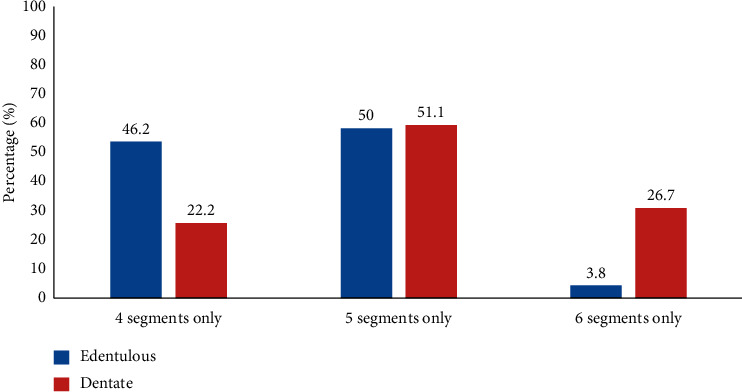
Percentage of edentulous and dentate mandibles with different numbers of segments.

Figure 2 shows the number of segments in the interforaminal distance in edentulous and dentate mandibles. In 12 (46.2%) of the edentulous mandibles and 10 (22.2%) of dentate mandibles, the interforaminal distance included only 4 segments. Only one (3.8%) edentulous mandible had six segments compared to 12 (26.7%) dentate mandibles. The mean (SD) interforaminal distance in edentulous mandibles was 45.77 (5.78) mm, significantly less than in dentate mandibles which was 50.44 (7.06) mm, *P*=0.006.

Table 1 shows that in segments 1–5, the mean mandibular thickness at level *A* in edentulous patients was less than in dentate patients. This difference was significant in segments 1–4 (*P* < 0.0001, <0.0001, 0.003, and 0.001). In segment 6, the thickness in one edentulous patient was greater than in dentate patients (mean = 8.90 and 7.05 mm), although this difference was statistically insignificant (*P*=0.37).

In anterior segments 2 and 3, in both levels *B* and *C*, the mean thickness in edentulous patients was significantly greater than in dentate patients (*P*=0.048, 0.01, 0.02, and 0.03). In the posterior segments, 1, 4, and 5, the thickness at levels *B* and *C* was less in edentulous than in dentate patients (*P*=0.25 and 0.36 in segment 1, *P*=0.80 and 0.47 in segment 4, and *P*=0.26 and 0.009 in segment 5). Table 1 also shows the difference between edentulous and dentate patients in mandibular height. Significantly shorter mandibles were observed in edentulous than in dentate patients in segments 1–5 (*P* < 0.0001) and in segment 6 where the difference was insignificant (*P*=0.22).

Table 2 shows that at level *A*, 25% of the edentulous mandibles had thickness <6 mm in segments 1–5 and 50% had thickness <6 mm in the anterior segments (2 and 3). More apically at level *B*, the thickness of edentulous mandibles was >6 mm in all segments in 100% of cases.

The lingual foramen was undetected in 7 mandibles (1 edentulous and 6 dentate) and was detected in 64 mandibles (90.1%).  Table 3 shows that significantly shorter distance from the base of the lingual canal to the alveolar crest was observed in edentulous patients than dentate patients (mean = 15.00 and 20.79 mm, *P* < 0.0001). No significant differences were observed between groups in the distance from the base of the lingual canal to the base of the mandible (*P*=0.16) or the length of the canal (*P*=0.82).

Table 4 shows that in edentulous and dentate groups, men had significantly greater mandibular height than women (*P*=0.02 and 0.005). In addition, men with edentulous mandibles had significantly greater distance between the base of the lingual canal and the alveolar crest than women (*P*=0.02), whereas in dentate patients, this difference was not statistically significant (*P*=0.19).

The 25^th^ lowest percentile of the distance between the base of the lingual canal and the alveolar crest was 12.3 mm indicating that in most edentulous patients, this distance was adequate to accommodate a 7 mm long implant. In only one patient, the distance was too short 6.2 mm.

## 4. Discussion

In this study, morphological features in the mandibular interforaminal region, the presence of lingual foramen and canal dimensions in dentate and edentulous Saudi patients, and its influence on future implant planning were evaluated.

The results showed that differences exist between edentulous and dentate patients regarding the interforaminal distance, height, and lingual canal location.

Considering the alveolar crest thickness, implant placement in the posterior interforaminal segments would be safe for 75% of edentulous patients, whereas placing implants in the anterior segments may not be safe for 50%. Thus, mandibular thickness at this level is critical for determining the safe zone where implants can be placed anterior to the mental foramina. Neither the position of the lingual canal nor mandibular height represented a constraint to implant placement in the anterior region of edentulous mandibles in the present study.

The present findings demonstrated an interforaminal distance of 50 mm in dentate and 46 mm in edentulous patients. Similarly, Quirynen et al. [22] reported a distance of 43–47 for different bone types, and Parnia et al. [23] found a distance of 50.8 mm in partially edentulous patients in addition to Madrigal et al. [8] who indicated that the distance among partially and completely edentulous patients was 46.5 mm. Variation in interforaminal distance may result from variations in the anatomical location of the mental foramen. In some individuals, the mental foramen is below the second premolar, while in others, it is between the first and second premolars [24].

In the present study, the mandible was significantly shorter in edentulous than dentate patients and in women than men. This agrees with a previous study that included 100 CBCTs of partially edentulous and dentate mandibles that reported significantly greater ridge height in males than females [25] and with Watanabe et al. [26] who reported significantly higher mandible in males than females. Similarly, Bulut and Köse [27] reported greater mandibular height in males than females, but the difference was statistically insignificant. Merrot et al. [28] reported less symphysis height among edentulous than dentate patients. On the other hand, Imirzalioglu et al. [29] assessed residual ridge resorption at the mental foramen area using panoramic radiographs and observed no significant difference between genders, although bone resorption was greater in females than males older than 50 years of age. In the present study, a significant difference between genders was observed in mandibular height in both dentate and edentate patients. This could be due to the continuous remodeling of the alveolar process. However, the resorption process is significantly greater in females than males after menopause [30]. Despite the alveolar process resorption throughout life, the basal bone (apical to the mental foramen) remains comparatively unchanged [30, 31]. The least bone height reported in the present study was 21 mm which is sufficient for placing dental implant of average length.

The current results showed that the mandible was slightly thicker in men than women with no significant difference in either dentate or edentulous patients. This comes in agreement with previous research reporting no significant difference between genders [26]. Similarly, Bulut and Köse [27] reported significantly greater bone width in males than females.

In the present study, 50% of the edentulous patients showed alveolar crest thickness less than 6 mm in the anterior segments. The minimal required thickness of alveolar bone is 5-6 mm to allow the insertion of dental implants that vary between 3 mm and 5 mm in diameter [32]. Accordingly, segments 1 and 4 were the most favorable for dental implant placement without augmentation, whereas 50% of edentulous patients might require ridge augmentation at the crestal region anteriorly to allow implant placement. These conclusions, however, are limited by the unavailability of data about time since edentulousness which might have affected the resorption of the mandible with subsequent reduced thickness. In other situations when time since teeth loss is shorter, less resorption might occur and the thickness might be adequate to support implant placement.

In the current study, the lingual foramen was detected in 90% of the images. Similar to Sener et al. [14], they reported the presence of lingual foramen in 95% of edentulous mandibles they examined. While Makris et al. [9] observed the lingual foramen in 81% of CBCTs and Pania et al. [23] in 49% of the examined CBCT scans.

The average length of the lingual canal in edentulous and dentate mandibles in the present study was 8.15 mm and 8.25 mm, respectively. Soto et al. [13] reported a 5 mm length of the lingual canal regardless of the dentition state. The distance between the base of the lingual canal and the alveolar crest was significantly lower in edentulous than dentate mandibles possibly due to resorption of the alveolar bone that follows tooth loss. However, this distance was 15 mm in edentulous mandibles which is adequate for placing endosseous implant. On the other hand, the average distance between the base of the lingual canal and the base of the mandible was 7.60 mm and 8.49 mm in edentulous and in dentate patients, respectively. These values are similar to those reported by Pania et al. [23] but less than those reported by Makris et al. [9].

For safe implant placement, no specific distance from the mental foramen was standardized. This could be attributed to anatomical variations including anterior loop length and diameters of incisive canal and lingual foramen that would require investigating each case separately [19, 23]. However, the present study suggests that segments 1 and 4 may show favorable anatomical features to accommodate dental implant in most patients.

The limitation of the present study is the unavailability of data regarding the duration of tooth loss, which might have affected bone resorption, and consequently, the observed differences between edentulous and dentate patient. In addition, the study included only one dental hospital which may limit the generalization to all Saudi Arabia. Cross-sectional studies with larger samples are recommended to assess the anatomic variations in edentulous and dentate mandibles that influence prosthetic rehabilitation.

## 5. Conclusions

Half of edentulous patients examined may receive normal size implants in the anterior interforaminal segments, while the other half will be limited to implants ≤3.5 mm or ridge augmentation procedure to allow standard implant placement. Neither the position of the lingual canal nor the mandibular height represented a constraint to implant placement in the anterior region of edentulous mandibles in the studied sample.

## Figures and Tables

**Table 1:** Difference between dentate and edentulous patients in mandibular height in mm and mandibular thickness at various levels.

Segment	Level	Edentulous patients (group I), *N* = 26		Dentate patients (group II), *N* = 45		*P* value
		Mean	SD	Mean	SD	
Segment 1 thickness	*A*	6.70	2.13	8.88	2.09	<0.0001 ^*∗*^
	*B*	8.92	2.01	9.53	2.16	0.25
	*C*	9.96	1.68	10.54	2.89	0.36
Segment 1 height		23.89	5.30	29.88	4.66	<0.0001 ^*∗*^
Segment 2 thickness	*A*	5.85	1.60	7.50	1.80	<0.0001 ^*∗*^
	*B*	9.01	2.44	7.95	1.93	0.048 ^*∗*^
	*C*	11.24	2.38	9.57	2.64	0.01 ^*∗*^
Segment 2 height		23.22	4.60	30.14	5.56	<0.0001 ^*∗*^
Segment 3 thickness	*A*	6.09	2.05	7.50	1.71	0.003 ^*∗*^
	*B*	8.88	2.26	7.65	1.94	0.02 ^*∗*^
	*C*	10.62	1.98	9.32	2.56	0.03 ^*∗*^
Segment 3 height		23.11	4.65	30.48	5.33	<0.0001 ^*∗*^
Segment 4^a^ thickness	*A*	6.47	2.15	8.28	1.60	0.001 ^*∗*^
	*B*	9.35	1.85	9.22	2.11	0.80
	*C*	10.11	2.38	10.61	2.89	0.47
Segment 4 height		22.92	5.12	30.68	4.74	<0.0001 ^*∗*^
Segment 5^b^ thickness	*A*	7.08	2.13	8.49	2.82	0.10
	*B*	9.49	1.74	10.33	2.51	0.26
	*C*	9.64	2.24	12.12	3.08	0.009 ^*∗*^
Segment 5 height		22.09	5.84	30.29	5.15	<0.0001 ^*∗*^
Segment 6^c^ thickness	*A*	8.90	.	7.05	1.90	0.37
	*B*	12.80	.	9.53	1.78	0.11
	*C*	11.90	.	11.07	2.88	0.79
Segment 6 height		21.50	—	30.17	6.36	0.22

^a^Number of edentulous and dentate patients = 26 and 45; ^b^*n* = 14 and 35; ^c^*n* = 1 and 11. ^*∗*^Statistically significant at *P* < 0.05.

**Table 2:** Thickness of mandible at levels *A* and *B* in edentulous patients.

Percentiles	Segment 1	Segment 2	Segment 3	Segment 4	Segment 5	Segment 6
Level *A*						
25^th^	4.95	4.40	4.66	4.65	5.13	8.90
50^th^	6.15	5.75	5.50	6.60	7.40	8.90
75^th^	8.10	7.15	7.70	8.80	8.80	8.90
Level *B*						
25^th^	7.38	7.18	7.00	8.10	7.88	12.80
50^th^	8.85	8.70	8.10	9.60	10.05	12.80
75^th^	10.63	10.45	10.95	10.70	11.08	12.80

**Table 3:** Comparison between edentulous and dentate patient regarding lingual canal features.

Lingual canal features	Edentulous patients (group I), mean (SD)	Dentate patients (group II), mean (SD)	*P* value
Distance from the base of the lingual canal to alveolar crest	15.00 (4.11)	20.79 (5.84)	<0.0001 ^*∗*^
Distance from the base of the lingual canal to the base of mandible	7.60 (2.16)	8.49 (2.62)	0.16
Length of canal	8.15 (2.06)	8.28 (2.02)	0.82

^*∗*^Statistically significant at *P* < 0.05.

**Table 4:** Gender difference between mandibular features in edentulous and dentate patients.

	Edentulous patients, mean (SD)		*P* value	Dentate, mean (SD)		*P* value
	Men	Women		Men	Women	
Mandibular thickness (mean of thickness at levels *A, B*, and *C*)	9.00 (1.47)	8.36 (1.80)	0.33	9.21 (2.34)	9.03 (1.21)	0.78
Mandibular height	25.24 (4.51)	21.02 (4.02)	0.02 ^*∗*^	32.55 (2.61)	28.89 (4.45)	0.005 ^*∗*^
Distance from the base of the lingual canal to alveolar crest	17.00 (3.60)	13.15 (3.76)	0.02 ^*∗*^	22.34 (6.45)	19.67 (5.41)	0.19
Distance from the base of the lingual canal to the base of mandible	8.15 (2.07)	7.10 (2.20)	0.23	8.62 (2.33)	8.31 (2.83)	0.73
Length of canal	8.56 (2.78)	7.78 (1.04)	0.38	8.19 (1.85)	8.26 (2.16)	0.92

^*∗*^Statistically significant at *P* < 0.05.

## Data Availability

The data used to support the findings of this study are available from the corresponding author upon request.
